# A Screen for Suppressors of Gross Chromosomal Rearrangements Identifies a Conserved Role for PLP in Preventing DNA Lesions

**DOI:** 10.1371/journal.pgen.0030134

**Published:** 2007-08-10

**Authors:** Pamela Kanellis, Mark Gagliardi, Judit P Banath, Rachel K Szilard, Shinichiro Nakada, Sarah Galicia, Frederic D Sweeney, Diane C Cabelof, Peggy L Olive, Daniel Durocher

**Affiliations:** 1 Samuel Lunenfeld Research Institute, Mount Sinai Hospital, Toronto, Ontario, Canada; 2 Department of Medical Genetics and Microbiology, University of Toronto, Toronto, Ontario, Canada; 3 British Columbia Cancer Research Centre, Vancouver, British Columbia, Canada; 4 Karmanos Cancer Institute, Detroit, Michigan, United States of America; National Institute of Diabetes and Digestive and Kidney Diseases, United States of America

## Abstract

Genome instability is a hallmark of cancer cells. One class of genome aberrations prevalent in tumor cells is termed gross chromosomal rearrangements (GCRs). GCRs comprise chromosome translocations, amplifications, inversions, deletion of whole chromosome arms, and interstitial deletions. Here, we report the results of a genome-wide screen in Saccharomyces cerevisiae aimed at identifying novel suppressors of GCR formation. The most potent novel GCR suppressor identified is *BUD16,* the gene coding for yeast pyridoxal kinase (Pdxk), a key enzyme in the metabolism of pyridoxal 5′ phosphate (PLP), the biologically active form of vitamin B6. We show that Pdxk potently suppresses GCR events by curtailing the appearance of DNA lesions during the cell cycle. We also show that pharmacological inhibition of Pdxk in human cells leads to the production of DSBs and activation of the DNA damage checkpoint. Finally, our evidence suggests that PLP deficiency threatens genome integrity, most likely via its role in dTMP biosynthesis, as Pdxk-deficient cells accumulate uracil in their nuclear DNA and are sensitive to inhibition of ribonucleotide reductase. Since Pdxk links diet to genome stability, our work supports the hypothesis that dietary micronutrients reduce cancer risk by curtailing the accumulation of DNA damage and suggests that micronutrient depletion could be part of a defense mechanism against hyperproliferation.

## Author Summary

Cells must ensure the integrity of genetic information before cellular division. Loss of genome integrity is particularly germane to tumorigenesis, where it is thought to contribute to the rapid evolution of the malignant cell towards the fully cancerous phenotype. It is therefore imperative that we understand fully how cells maintain the integrity of the genome and how it is lost during tumorigenesis. In this study, we developed an assay that allowed us to systematically interrogate each gene of the budding yeast S. cerevisiae for its respective contribution to genome integrity. We report the identification of nine novel genes that increase the rate of genome instability in yeast when deleted. To our surprise, one of the genes we identified encodes the enzyme pyridoxal kinase, which acts in the metabolism of vitamin B6. We show that pyridoxal kinase influences genome stability by promoting the conversion of dietary vitamin B6 into its biologically active form, pyridoxal 5′ phosphate. Our work indicates that vitamin B6 metabolites are critical to maintain genome stability and supports a long-standing model, which hypothesizes that vitamin B6 reduces cancer risk by curtailing genome rearrangements.

## Introduction

The faithful replication of the genome is necessary for maintenance of genome integrity. Disrupting processes that ensure faithful DNA replication results in chromosome breakage, hyper-recombination, or gross chromosomal rearrangements (GCRs) [1–3]. This relationship has been particularly highlighted in the budding yeast *S. cerevisiae,* where GCRs arise at high rates in cells with defects in the S-phase checkpoint [4], DNA replication licensing [5,6], DNA replication elongation [7–9], chromatin assembly [10], and homologous recombination (HR) repair [8].

Altogether, these studies not only suggest a common origin (i.e., DNA replication), but also a common mechanism by which genome rearrangements are formed [2]. Defects that occur during DNA replication lead to elevated levels of DNA damage, including DNA double-strand breaks (DSBs). In turn, these lesions may serve as substrates for the illegitimate repair processes resulting in GCRs. Therefore, identification of genes that prevent GCRs can potentially uncover novel genome caretakers that guard cells against the accumulation of mutations. In addition, unbiased identification of GCR suppressors could be a useful route for discovering novel genes and pathways that participate in DNA replication.

**Figure 1:**
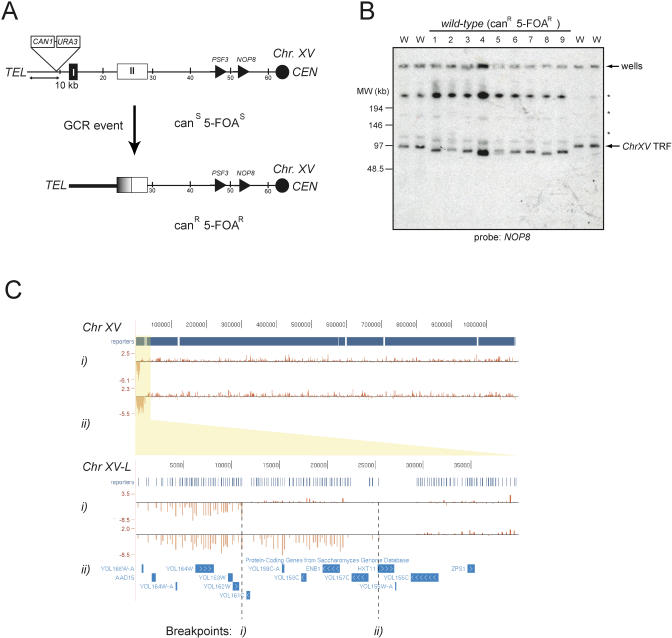
The *ChrXV-L* GCR Assay (A) Schematic of the *ChrXV-L* GCR reporter chromosome. A GCR event in this region of *ChrXV-L* can result in the loss of the *URA3* and *CAN1* genes, which yields a canavanine and 5-FOA resistant strain (can^R^ 5-FOA^R^). *PSF3* is the first essential gene on *ChrXV-L*. HRI and HRII denote two regions of homology that are centered around the *PAU20* and *HXT11* genes, respectively. (B) PFGE analysis of *ChrXV-L* terminal restriction fragments following PmeI digestion of genomic DNA isolated from either the parent strain (W) or strains that have undergone a GCR event (1–9). Asterisk indicates incomplete digestion products of *ChrXV*. (C) Array-based comparative genome hybridization of two strains that have undergone a GCR event at *ChrXV-L*. The above panel is a histogram representation of log_2_-tranformed relative signal enrichments on Chromosome *XV* viewed in the University of California Santa Cruz genome browser [69]. The location of the array probes (reporters) is also indicated. Note the large loss of sequences on the left arm of *ChrXV* in both strains. The lower panel zooms to the *ChrXV-L* subtelomeric region. The breakpoint in strain *(i)* must be in the vicinity of the *PAU20* gene *(YOL161C),* whereas that of strain *(ii)* resides in the vicinity of *HXT11*.

Most of the current knowledge regarding GCR formation originates from candidate gene studies examining rearrangements at a single locus in budding yeast, the left arm of Chromosome *V* (*ChrV-L*). Although this locus has been instrumental in the deciphering of many basic mechanisms governing genome stability in eukaryotes, examination of GCR formation at other loci provides a complementary view. For example, the use of yeast artificial chromosomes to study GCRs led to the discovery that defective chromosome condensation (in a *ycs4* mutant) results in GCR events [7]. In addition, studies employing a Chromosome *VII* disome found that defects in DNA replication and checkpoint control elevate rates of chromosome loss and rearrangements following replication fork stalling [11]. In another study, Hackett et al. employed the telomeric region of *ChrXV-L* to study GCR events triggered by telomerase dysfunction [12]. This latter locus is particularly useful since GCRs at *ChrXV-L* involve break-induced replication (BIR), a type of homologous recombination repair predicted to be a major source of genome rearrangements [2,13–15]. In contrast, GCRs formed at *ChrV-L* are primarily the consequence of de novo telomere addition [8]. This difference can be explained by the architecture of the telomere-proximal region on *ChrXV-L,* which contains two regions of homology (HRI centered on the *PAU20* gene, and HRII centered on the *HXT11* gene; Figure 1A) located 12 kb and 25 kb from the telomere [12]. These regions share a high degree of sequence identity with other regions in the genome [12]. As a consequence, DNA lesions formed at loci telomeric to HRI or HRII are predominantly repaired by BIR, producing nonreciprocal translocations in haploid cells. Notably, increased repair by BIR can also lead to loss of heterozygosity in diploid genomes, which may accelerate the process of tumorigenesis by inactivation of tumor suppressor genes.

In this study, we screened the yeast genome for mutants that increase the level of chromosome rearrangements; specifically, those that increase the frequency of BIR-mediated nonreciprocal translocations. We report the construction of a strain containing a GCR reporter on *ChrXV-L* that is amenable to genome-wide screening and compatible with synthetic genetic array technology [16]. We employed this strain to systematically screen the gene deletion collection [17] leading to the identification of nine new GCR suppressors. Here, we focus on the characterization of one of the most potent GCR suppressors identified, *BUD16,* which encodes yeast pyridoxal kinase (Pdxk), a critical enzyme in vitamin B6 metabolism. We show that Pdxk is critical for the maintenance of genome integrity via its role in maintaining adequate levels of pyridoxal 5′ phosphate (PLP), the biologically active form of vitamin B6. Our results are consistent with a model whereby dTMP biosynthesis is the pathway affected by a decrease in PLP, thus providing an important link between dietary micronutrients, DNA replication and genome stability. Furthermore, since many epidemiological studies have linked defective vitamin B6 levels to an increased cancer incidence [18–23], our study supports the hypothesis that micronutrients such as vitamin B6 curtails carcinogenesis by preventing genomic instability.

## Results

### A System for the Facile Recovery of BIR-Mediated GCR Events

To generate a GCR reporter strain that is amenable to genome-wide screening, we adapted a system previously described by Hackett et al. [12]. We inserted the *CAN1* and *URA3* genes, two counter-selectable markers, ∼10 kb from the telomere of *ChrXV-L* (Figure 1A). The simultaneous loss of *CAN1* and *URA3* (detected on media containing canavinine [can] and 5-fluoro-orotic acid [5-FOA]) at this locus occurs at a rate of 8.9 × 10^−8^ (Table 1), approximately 250-fold higher than the rate observed at *ChrV-L* (3.5 × 10^−10^; Table 2). This elevated GCR rate may be due to the higher efficiency of BIR over de novo telomere addition in repairing DSBs. Moreover, the HRI and HRII regions on *ChrXV-L* display between 85%–97% homology with a total of 21 chromosome arms [12]. This large number of potential seeds for BIR may also explain the relatively high GCR rate at *ChrXV-L*. To ensure that the GCR events recovered from the simultaneous loss of *CAN1* and *URA3* are due to BIR, we analyzed GCR events in wild-type cells by pulsed-field gel electrophoresis (PFGE) using a scheme described by Hackett et al. [12]. Briefly, we isolated genomic DNA from parental can^S^ 5-FOA^S^ cells and cells that have undergone GCR events at *ChrXV-L* (can^R^ 5-FOA^R^ cells). This DNA is then digested with PmeI to liberate a terminal restriction fragment, separated by PFGE, and finally transferred onto nitrocellulose by Southern blotting to be probed with a *ChrXV-L*-specific fragment (*NOP8;* Figure 1B). If BIR occurs by employing any of the 21 homologous chromosome arms as a template, the resulting terminal restriction fragments liberated by PmeI are all predicted to be of lower molecular weight than the parent fragment (∼97 kb). As predicted, the analysis of nine can^R^ 5-FOA^R^ mutants derived from the wild-type strain indicate that nine out of nine have undergone a GCR event at *ChrXV-L* that is consistent with BIR, since their terminal restriction fragments all migrate faster than that of the parental strain (Figure 1B). Furthermore, the analysis of four can^R^ 5-FOA^R^ strains by comparative genome hybridization using tiling microarrays identified breakpoints either in HRI, in the vicinity of the *PAU20* gene (in two of four strains analyzed), or in HRII (i.e., in the vicinity of the *HXT11* genes, two of four strains; see Figure 1C for two representative examples). Lastly, we determined the GCR rate at *ChrXV-L* of a *rad52*Δ strain, since *RAD52* is required for BIR. The *rad52*Δ strain does not produce any detectable GCR events under the standard conditions of our assay (i.e., the rate must be ≪8.4 × 10^−9^; Table 2). This result suggests that most of GCR events observed at *ChrXV-L* are indeed dependent on *RAD52,* a gene required for BIR. Collectively, the above results indicate that the *ChrXV-L* GCR reporter monitors BIR-type events.

**Table 1:**
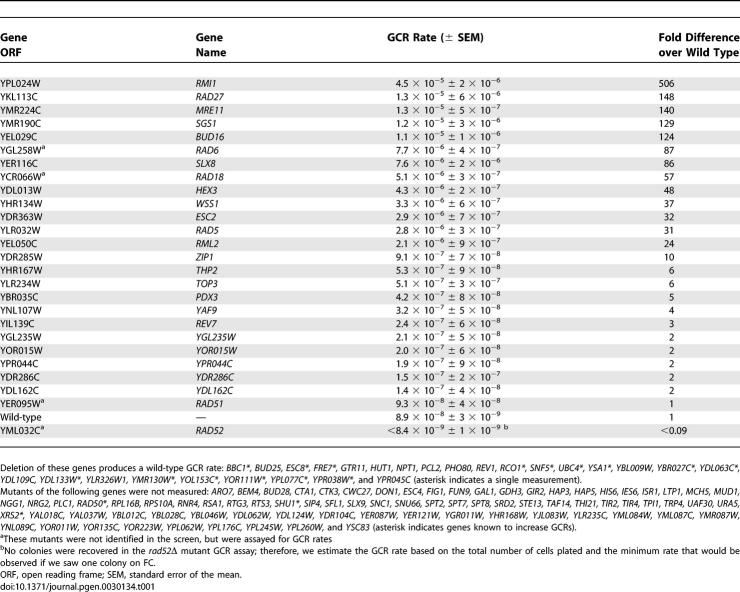
GCR Rates of Mutants Identified from the Genome-Wide GCR Papillation Assay

**Table 2:**
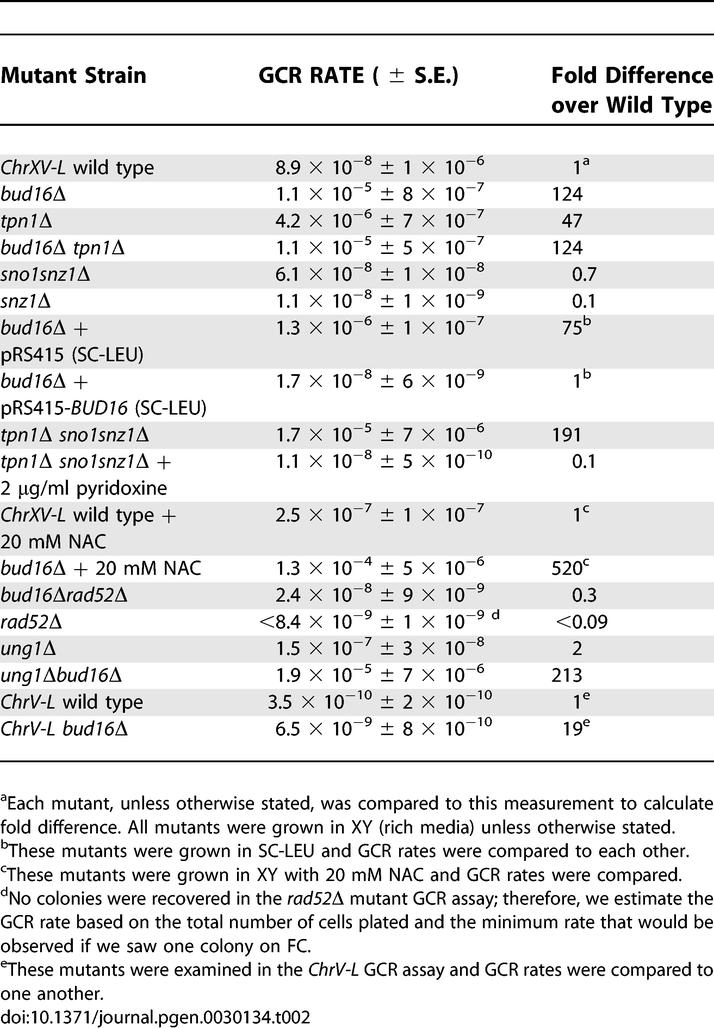
*ChrXV-L* and *ChrV-L* GCR Rates of Vitamin B6 Mutants

### A Genome-Wide Screen for Suppressors of BIR-Mediated GCR Events

**Figure 2:**
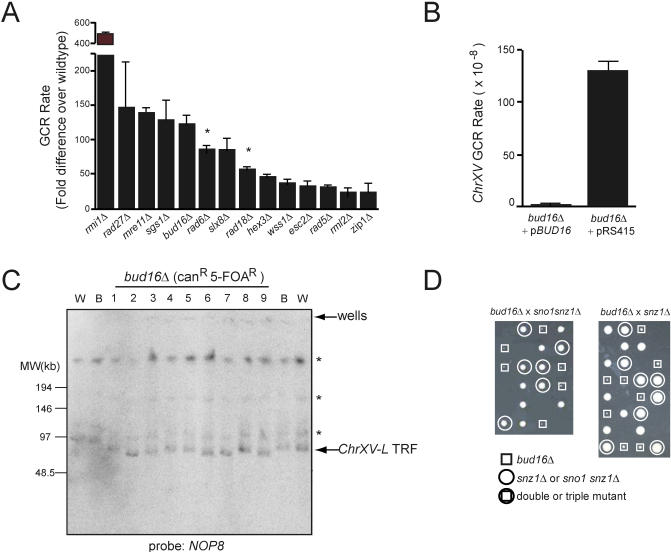
A Genome-Wide Screen Identifies *BUD16* as a Key Determinant for Genome Stability in S. cerevisiae (A) GCR rates at *ChrXV-L* of the indicated strains. The data is presented as the fold-increase over the wild-type GCR rate +/− strandard error of the mean. Asterisk refers to genes with high GCR rates that were not identified in the screen, but were measured during the course of this study. (B) A plasmid encoding *BUD16* suppresses the high GCR rate of *bud16*Δ. (C) PFGE analysis of *ChrXV-L* terminal restriction fragments following PmeI digestion of genomic DNA isolated from either the wild-type (W), parent *bud16*Δ strains (B), or *bud16*Δ strains that have undergone a GCR event (1–8). Asterisk indicates incomplete digestion products of *ChrXV*. (D) The *bud16*Δ mutation is synthetic lethal with the deletions of *SNO1* and *SNZ1*.

We crossed the resulting *ChrXV-L* GCR assay strain with the 4,812 viable open reading frame deletion strains [16,17] and employed a semi-quantitative papillation assay to monitor GCR formation (Figure S1). An initial set of 48 strains that scored positive were reconstructed in the *ChrXV-L* assay strain to determine their GCR rate by fluctuation analysis [24] (Table 1). This group included deletions in several known GCR suppressors such as the genes encoding Mre11, a component of the MRX complex, the RecQ helicase Sgs1, and the budding yeast FEN-1 homolog, Rad27. Using this scheme, we identified nine gene deletions that display at least a 10-fold increase in GCR rate compared to wild type (Table 1 and Figure 2A). Of these nine novel GCR suppressors, mutations in *RMI1, RAD5, SLX8,* and *HEX3* were independently reported during the course of this study to promote GCRs at *ChrV* [25–27].

The remaining five novel GCR suppressors include *BUD16*, *WSS1, ESC2, RML2*, and *ZIP1*. Intriguingly, *ZIP1* encodes a component of the synaptonemal complex that is active during meiosis [28] and is also expressed in mitotic cells [29], suggesting a potential role for Zip1 in mitotic genome stability. *RML2* encodes the mitochondrial L2 ribosomal protein [30]. Surprisingly, a Rml2-GFP fusion protein localizes to the nucleus [31], suggesting a putative nuclear function for Rml2. *WSS1* encodes a weak suppressor of an *smt3* mutation [32], and *ESC2* encodes a protein harboring a SUMO domain that has been linked to heterochromatic silencing [33,34] and the function of the Smc5/6 complex [35]. BLAST searches and alignments reveal that *BUD16* encodes a putative Pdxk. With a GCR rate of 1.1 × 10^−5^ (124-fold above wild type), *bud16*Δ is within the range of very potent GCR mutator deletions that include *rad27*Δ (1.3 × 10^−5^; 148-fold over wild-type rate), *mre11*Δ (1.3 × 10^−5^; 140-fold), and *sgs1*Δ (1.2 × 10^−5^; 129-fold) (Figure 2A and Table 1). Reintroduction of a plasmid encoding wild-type *BUD16* complemented the genome instability of *bud16*Δ cells, eliminating the possibility that a second site mutation contributes to its elevated GCR rate (Figure 2B). We also examined the type of GCR events promoted by the *bud16*Δ mutation by PFGE, which indicated that *BUD16* prevents BIR-type rearrangements at *ChrXV-L* (Figure 2C). Given that *bud16*Δ had the most profound effect on genome stability among the uncharacterized suppressors, we focused on deciphering its role in preventing chromosome rearrangements.

### BUD16 Encodes the Yeast Pyridoxal Kinase

In all organisms, Pdxk is an essential component of a vitamin B6 salvage pathway that ultimately produces PLP [36]. To ascertain whether *BUD16* functions as the budding yeast Pdxk, we measured PLP levels in wild-type and *bud16*Δ strains. We found that the PLP levels of *bud16*Δ cells are only 1.8% of wild-type levels (Table 3). This result is somewhat surprising, since bacteria, yeast, and plants also possess a de novo vitamin B6 pathway that produces PLP in a Pdxk-independent manner. In yeast, this pathway is under the control of the *SNO1* and *SNZ1* genes [37]. However, these genes are not normally expressed during logarithmic growth but rather are expressed during stationary phase or under poor nutrient conditions. We found that the simultaneous deletion of the *SNO1* and *SNZ1* locus did not significantly reduce PLP levels (95.2% of wild-type levels; Table 3) or increase GCR rates (Table 2). Although *SNO1* and *SNZ1* deletion did not significantly impact genome stability (or PLP levels) in *BUD16* cells, the de novo vitamin B6 synthetic pathway is nevertheless essential for viability in the absence of *BUD16*. Indeed, we are unable to recover viable triple mutants from a cross between *bud16*Δ and *sno1snz1*Δ or double mutants from a cross between *bud16*Δ and *snz1*Δ (Figure 2D). Overall, the decrease in intracellular PLP levels in *bud16*Δ along with its synthetic lethality with *sno1snz1*Δ are consistent with the idea that *BUD16* functions in parallel with the de novo B6 pathway as a yeast pyridoxal kinase. Additional characterization of the *bud16*Δ strain in terms of growth and cell cycle kinetics is described in Text S1 and in Figure S2 and Tables S1 and S2.

**Table 3:**
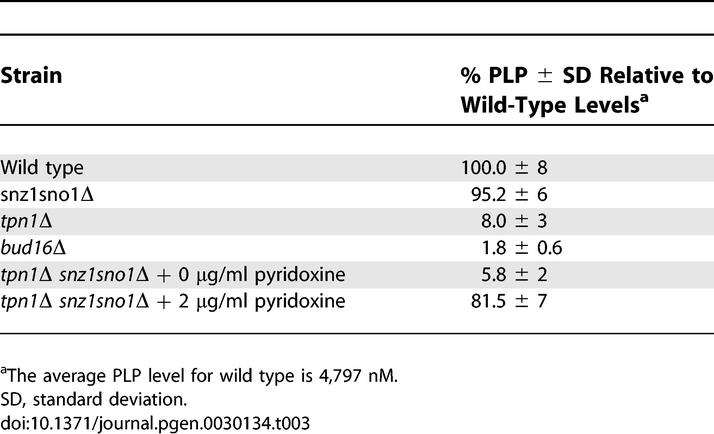
Levels of Vitamin B6 (PLP) Measured by HPLC

### BUD16 Prevents Genomic Instability at Multiple Genomic Loci

**Figure 3:**
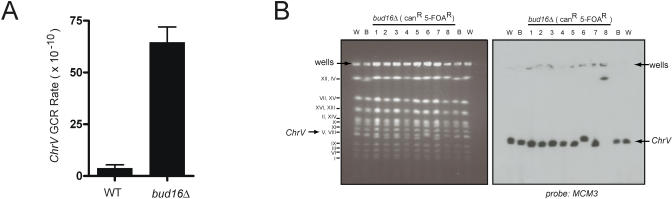
Deletion of *BUD16* Also Increases GCR Formation at *ChrV-L* (A) GCR rate of either the wild-type (WT) or *bud16*Δ strain at *ChrV-L*. (B) Whole-chromosome PFGE analysis of wild-type (W), parental *bud16*Δ (B), or *bud16*Δ strains that have undergone a GCR event (1–8). Left panel: ethidium bromide–stained gel. Right panel: Southern blot with a *ChrV*-specific probe *(MCM3).*

To determine whether the *bud16*Δ mutation increases genome instability across the genome, we calculated the GCR rate of a *bud16*Δ strain at the *ChrV-L* locus [8]. We found that the *bud16*Δ mutation elevates the GCR rate at this locus 19-fold over the wild-type rate (Figure 3A; Table 2). Analysis of the GCR events involving *ChrV* by whole-chromosome PFGE reveals a mixture of events consistent with de novo telomere additions (six out of eight events analyzed) and nonreciprocal translocations (two out of eight events) (Figure 3B). This ratio of telomere additions to translocations (4:1) is similar to the ratio of GCRs typically recovered from a wild-type strain [4]. Together, these results indicate that *BUD16* suppresses different types of genome rearrangements at a minimum of two different loci in the genome, suggesting that *BUD16* may act to prevent the occurrence of DNA lesions rather than by promoting a specific type of illegitimate repair.

**Figure 4:**
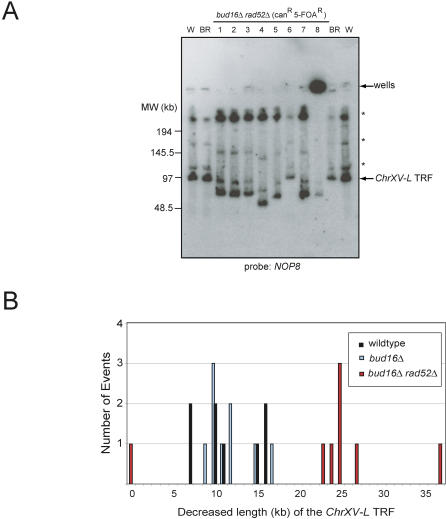
Homologous Recombination Is Required for the BIR-Mediated GCR Events in *bud16*Δ Cells (A) PFGE analysis of *ChrXV-L* terminal restriction fragments following PmeI digestion of genomic DNA isolated from either the wild-type (W), parent *bud16*Δ*rad52*Δ strains (BR), or *bud16*Δ*rad52*Δ strains that have undergone a GCR event (1–8). Asterisk indicates incomplete digestion products of *ChrXV*. An undigested Chromosome *XV* likely explains the presence of a signal in the well of strain 8, although a weak TRF signal can be detected. (B) Quantitation of the terminal restriction fragment length decrease following GCRs when the *Pme*I TRFs from *bud16*Δ and *bud16*Δ*rad52*Δ cells are compared to those of wild type. TRF, terminal restriction fragment.

To further characterize the mechanism that underlies the high GCR rate of *bud16*Δ cells in the *ChrXV-L* assay, we crossed the *bud16*Δ GCR reporter strain to a strain containing a deletion of *RAD52,* a gene necessary for all types of homologous recombination, including BIR [15]. The GCR rate at *ChrXV-L* of the *bud16*Δ *rad52*Δ double mutant was reduced to wild-type levels (2.4 × 10^−8^; Table 2). However, this rate is far greater than the GCR rate of a *rad52*Δ mutation alone (<<8.4 × 10^−9^; Table 2). Furthermore, analysis of the terminal restriction fragment of the rearranged chromosomes in the *bud16*Δ *rad52*Δ double mutant shows terminal deletions in seven out of eight cases that are strikingly larger than those observed in either wild-type or *bud16*Δ strains (23–37 kb shorter in *rad52*Δ strains versus ∼7–17 kb in the *RAD52* ^+^ strains; Figure 4A and 4B). This difference in the size of the *ChrXV-L* terminal restriction fragment suggests that *bud16*Δ *rad52*Δ do not undergo BIR-mediated GCR events that employ the HRI/II regions as seeds. Instead, in the absence of functional HR, these mutants are likely repaired by de novo telomere additions, leading to large terminal deletions.

### Elevated Levels of DNA Lesions and Activation of the DNA Damage Checkpoint in *bud16*Δ Cells

**Figure 5:**
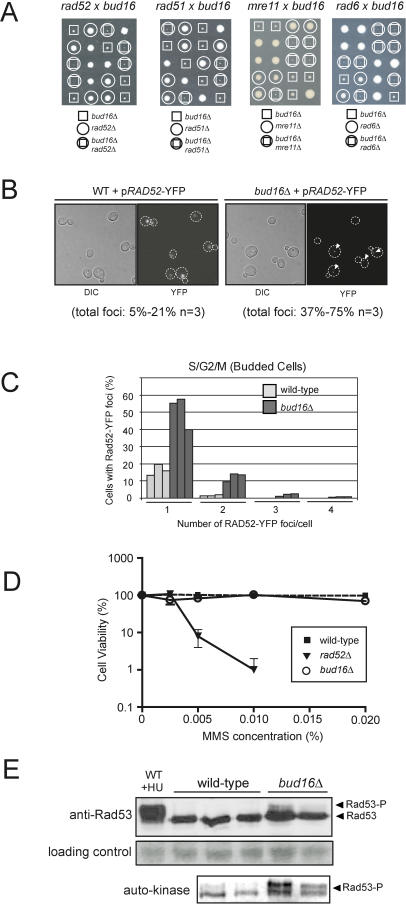
The *BUD16* Gene Deletion Causes DNA Lesions and Checkpoint Activation (A) Tetrad analysis of crosses between *bud16*Δ and *rad52*Δ, *rad51*Δ, *mre11*Δ, or *rad6*Δ. (B) Micrographs of wild-type (WT) or *bud16*Δ cells expressing Rad52-YFP. Left panels: differential interference contrast (DIC). Right panels: YFP fluorescence microscopy (YFP). Arrowheads point to Rad52-YFP foci. (C) Quantitation of Rad52-YFP foci per cell. Three independent isolates were examined with over 180 cells per isolate counted. (D) Survival curves of wild-type, *bud16*Δ or *rad52*Δ cells serially diluted and plated onto rich media +/− MMS (as indicated) and grown for 3–8 d at 30 °C in triplicate. Percent survival at a given MMS concentration represents the number of colony-forming units of the indicated strain divided by the colony-forming units of the wild type plated on media lacking MMS. No viable *rad52*Δ colonies were recovered at concentrations above 0.015% MMS. (E) *bud16*Δ cells engage the DNA damage checkpoint. Upper panel: Rad53 immunoblots of extracts of the indicated strains. Middle panel: Ponceau stain for loading control. Lower panel: Rad53 activity assessed by auto-kinase assays [40]. The immunoblot and auto-kinase assays were performed on the same extracts.

Together, the observations that *BUD16* suppresses GCRs at multiple loci in a BIR-dependent and independent manner suggest that *bud16*Δ cells experience higher-than-normal levels of DNA lesions during vegetative growth. We find support for this possibility when tetrads from a cross between *bud16*Δ and *rad52*Δ are examined (Figure 5A). We observe that the *bud16*Δ *rad52*Δ double mutant displays synthetic sickness and poor viability when compared to their congenic single mutants. This result suggests that *bud16*Δ cells may experience high levels of genotoxic stress that require the HR pathway for optimum viability. Consistent with this explanation, the *bud16*Δ mutation also displays synthetic sickness when crossed with an *MRE11* gene deletion and to a lesser extent with deletion of *RAD51,* two additional genes acting in the homologous repair of DSBs (Figure 5A). We also observe a strong genetic interaction between the *bud16*Δ and *rad6*Δ mutations (Figure 5A), suggesting that DNA lesions caused by the reduction of PLP levels may require post-replicative repair or lesion bypass. Based on this spectrum of genetic interactions, *bud16*Δ cells likely accumulate DNA lesions during DNA replication, possibly leading to replication fork stalling or collapse.

To gain more direct evidence for the presence of active *RAD52*-dependent recombination in *bud16*Δ cells, we monitored the formation of Rad52 DNA repair centers [38]. Upon formation of lesions that engage HR, Rad52 relocalizes from a diffuse nuclear pattern into discernable punctate foci that coincide with DNA lesions. We thus expressed a Rad52 protein fused to the yellow fluorescent protein (YFP) in wild-type and *bud16*Δ cells and examined the presence of Rad52 repair centers by fluorescent microscopy (Figure 5B). In *bud16*Δ cultures, 37%–75% of the cells display Rad52-YFP foci compared to 5%–21% of wild-type cells (Figure 5B). In *bud16*Δ cultures, Rad52-YFP foci are surprisingly found in G1 (unbudded) cells but are most prevalent in S/G2/M (budded) cells (57%–75% in budded versus 37%–59% in unbudded cells). Intriguingly, the presence of Rad52 foci in G1 nuclei suggests the presence of persistent or unrepairable DNA lesions in cells that have undergone checkpoint adaptation [39]. Furthermore, we observe that budded *bud16*Δ cells display greater than one repair centre in 12–18% of the cells whereas this situation occurs in less than 2% of wild-type cells (Figure 5C). Since up to ten DSBs may localize to one repair centre [38], these results suggest that *bud16*Δ cells experience high levels of DNA lesions during DNA replication. Alternatively, we cannot exclude the possibility that *bud16*Δ cells have a dramatically reduced rate of DNA repair. However, *bud16*Δ cells are not sensitive to the radiomimetic alkylating agent methyl methanesulfonate (MMS; Figure 5D), indicating that the Rad52 pathway is functional in these cells. Therefore, the increased presence of Rad52 foci in *bud16*Δ cells is most likely explained by an increased number of DNA lesions.

Next, we examined whether the spontaneous DNA damage present in *bud16*Δ cells is sufficient to activate the DNA damage checkpoint pathway by assaying Rad53, the yeast homolog of the tumor suppressor Chk2. Rad53 kinase activation is observed by a detectable auto-kinase activity [40] concomitant with a reduced mobility on SDS-PAGE due to autophosphorylation. As shown in Figure 5E, Rad53 is hyperactivated in *bud16*Δ cells when compared to wild type, indicating that sufficient DNA damage is present in the *bud16*Δ mutant to activate the DNA damage checkpoint. Phosphorylation of Rad53 in cycling populations is often seen in strains that experience high levels of spontaneous DNA lesions, such as *dia2*Δ, *rrm3*Δ, and *rmi1*Δ, among others [25,41,42]. Altogether, our results are consistent with a model whereby *bud16*Δ cells experience high levels of DNA lesions, including DSBs. These DNA lesions most likely serve as substrates for illegitimate repair, resulting in elevated levels of genome rearrangements.

### Normal PLP Levels Are Required for Genome Integrity

**Figure 6:**
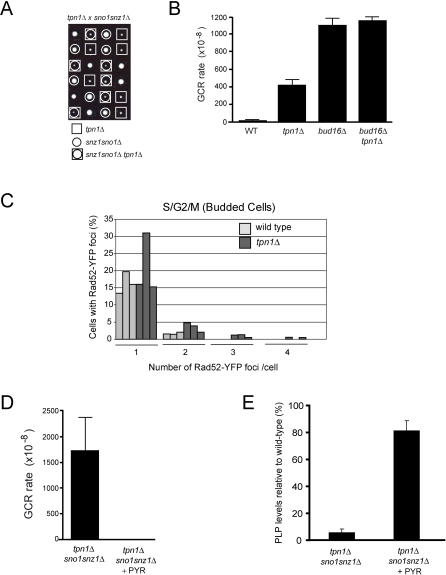
PLP Levels Correlate with Genome Stability (A) Tetrad analysis of *tpn1*Δ (square) crossed to *sno1snz1*Δ (circle). Viable triple mutant *tpn1*Δ *sno1snz1*Δ can be recovered (circle in square). (B) GCR rates at *ChrXV-L* of the indicated strains. (C) Quantitation of Rad52-YFP foci per cell in wild-type and *tpn1*Δ cells. (D) GCR rates at *ChrXV-L* of *tpn1*Δ *sno1snz1*Δ triple mutant grown with pyridoxine (+PYR) or without added pyridoxine to the media. (E) Relative PLP levels of the *tpn1*Δ *sno1snz1*Δ triple mutant grown with pyridoxine (+PYR) or without added pyridoxine to the media.

The genome instability observed in the *bud16*Δ mutant correlates with low levels of PLP. However, this observation does not eliminate the possibility that its GCR mutator phenotype could be due to a PLP-independent function of Bud16/Pdxk. To address this possibility, we aimed to reduce PLP levels by alternative means to probe the relationship between PLP and genome integrity. As a first means, we inactivated other components of the vitamin B6 salvage pathway. In particular, when yeast are grown to log phase under laboratory conditions, the PLP precursor, pyridoxine, is actively transported into cells mainly, but not solely, by the Tpn1 transporter [43]. Therefore, we asked whether *TPN1* deletion impacts total PLP levels and genome stability. Cells harboring a *tpn1*Δ mutation have low levels of intracellular PLP (8% of wild-type; Table 3) that are nevertheless higher than those of *bud16*Δ. At a genetic level, we find that the *tpn1*Δ mutation is not synthetic lethal with the *sno1snz1*Δ double mutant (Figure 6A). The continued viability of the *tpn1*Δ *sno1snz1*Δ mutant supports the observation that although Tpn1 is a component of the B6 salvage pathway, it is not absolutely essential for pyridoxine transport [43]. Accordingly, the GCR rate of the *tpn1*Δ strain at *ChrXV-L* is increased 47-fold over the wild-type rate, which is less that of the *bud16*Δ GCR rate (124-fold over wild type; Figure 6B). However, both genes act in the same pathway to suppress genome rearrangements, as the double *bud16*Δ *tpn1*Δ mutant display the same GCR rate as *bud16*Δ (Figure 6B). Lastly, we find multiple Rad52 recombination centers present in 4%–7% of *tpn1*Δ budded cells, suggesting the presence of catastrophic DNA damage similar to that seen in *bud16*Δ, albeit at a lower level (Figure 6C). Altogether, these results further suggest that PLP levels correlate with genome integrity.

In addition to manipulating PLP levels via genetic means, we also manipulated pyridoxine intake to further explore the link between PLP levels and genome integrity. To carry out these experiments, we disabled de novo vitamin B6 synthesis (via *SNO1 SNZ1* inactivation) to exclude the contribution of this pyridoxine-independent pathway. We also impaired pyridoxine transport by deleting *TPN1.* When grown in rich media, the resulting *tpn1*Δ *sno1snz1*Δ triple mutant has an elevated GCR rate (191-fold over wild type), which is greater than either the *tpn1*Δ or *sno1snz1*Δ mutants (Table 2). Accordingly, when we measure the PLP levels of this strain, we find that they are 5.8% of wild-type levels (Table 3). Importantly, we then supplemented the growth media of *tpn1*Δ *sno1snz1*Δ with 2 μg/ml pyridoxine as a means to stimulate its transport across the membrane. As shown in Figure 6D and Table 2, addition of pyridoxine to the media of *tpn1*Δ *sno1snz1*Δ potently suppresses its GCR rate to wild-type levels. Critically, under the same conditions, the PLP levels of the *tpn1*Δ *sno1snz1*Δ strain are dramatically increased to 81.5% of wild type (Figure 6E; Table 3). Together, these data conclusively demonstrate a relationship between PLP levels and maintenance of genome integrity.

### Pharmacological Inhibition of Pdxk in Human Cells Causes DNA DSBs

**Figure 7:**
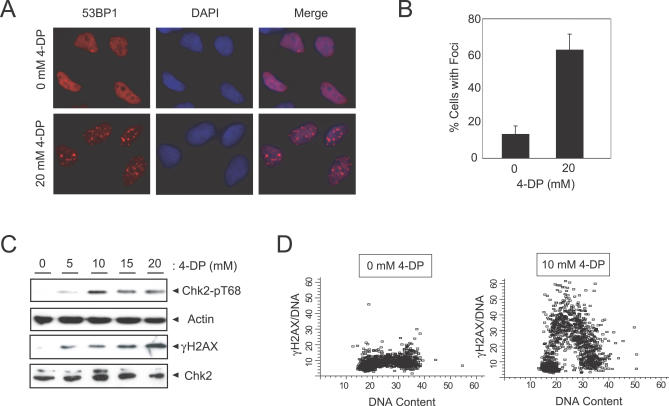
Inhibition of Pdxk Causes DSBs in Human Cells (A) HeLa cells display elevated levels of 53BP1 foci following inhibition of Pdxk by 4-DP. (B) Quantitation of cells with 53BP1 foci following 4-DP treatment. (C) 4-DP treatment activates the DNA damage checkpoint in HeLa cells as measured by Chk2 phospho-Thr68 and γ-H2AX immunoblotting. (D) SiHa cells treated with 4-DP accumulate γ-H2AX in S-phase, as measured by flow cytometry.

We next examined the role of Pdxk on the genome integrity of human cells by employing the well-characterized vitamin B6 analog 4-deoxypyridoxine (4-DP) [44]. First, we determined whether inhibition of human Pdxk leads to DNA damage, particularly DSBs. To detect DSBs in human cells, we examined the localization of 53BP1, a DNA repair and signaling protein, by immunofluorescence microscopy. 53BP1 forms nuclear foci that colocalize with DSBs in mammalian cells and is thus a useful surrogate marker of this type of DNA damage [45]. As shown in Figure 7A and 7B, addition of 4-DP to the media of HeLa cells causes an accumulation of 53BP1 foci. Second, we found that 4-DP treatment also triggers activation of the checkpoint kinase Chk2, as evidenced by phosphorylation of its Thr68 residue (Figure 7C). Third, we analyzed the phosphorylation status of H2AX on its C-terminal Ser139 residue (known as γ-H2AX), one of the earliest events in the response to DSBs. The presence of γ-H2AX was assessed via immunoblotting (Figure 7C) and flow cytometry (Figure 7D) [46]. In cells treated with the Pdxk inhibitor 4-DP, γ-H2AX clearly accumulates during S-phase between the 2N and 4N DNA content, similar to what is observed in yeast cells (with Rad52-YFP). Importantly, to ensure that the described effects were not due to apoptotic effects caused by Pdxk inhibition at the concentrations of 4-DP employed above, we measured levels of apoptosis in HeLa cells by annexin V staining (Figure S3). From this data, we can rule out the possibility that 4-DP triggers DSB formation via the activation of an apoptotic program. Instead, our results indicate that, as in yeast cells, Pdxk inhibition induces DNA lesions and activation of the DNA damage response.

### PLP May Affect dTMP Biosynthesis to Impact Genome Stability

**Figure 8:**
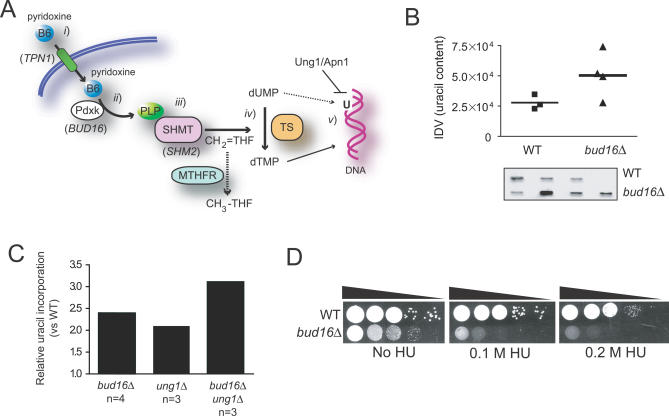
PLP levels Are Required for Optimal dTMP Biosynthesis (A) The intersection of vitamin B6 and dTMP biosynthesis. *(i)* Dietary vitamin B6 (i.e., pyridoxine) is imported into the cell via the Tpn1 transporter (S. cerevisiae gene names in brackets). *(ii)* Pdxk phosphorylates the B6 vitamers (pyridoxine, pyridoxamine, and pyridoxal) to generate PLP, which acts *(iii)* as a cofactor for serine hydroxymethyl transferase (SHMT). *(iv)* SHMT is necessary for the formation of methylenetetrahydrofolate (CH_2_=THF), the methyl group donor for the conversion of dUMP into dTMP. *(v)* A deficiency in PLP is predicted to reduce dTMP levels leading to a nucleotide pool imbalance and incorporation of uracil into DNA. (B) *bud16*Δ cells accumulate uracil in DNA, as measured by a modified aldehydic slot blot assay. The results of the slot blot (bottom panel) were quantified and shown in the graph. IDV refers to the integrated density values of the bands. (C) Uracil accumulation in the *bud16*Δ genomic DNA is comparable to that observed in *ung1*Δ DNA. (D) *bud16*Δ cells are sensitive to nucleotide depletion by hydroxyurea at 0.2 M. The results of a colony forming assay can also be found in Table S3.

We finally sought to narrow down the biological pathway in which PLP acts to promote genome stability. This is a difficult task, since PLP is a critical cofactor for numerous essential enzymes acting in amino acid and dTMP biosynthesis. However, our observations in yeast and human cells indicate a role for PLP in preventing DNA lesions during DNA replication, pointing to dTMP synthesis as the likeliest candidate pathway linking PLP to genome stability (Figure 8A). This possible association is strengthened by the multitude of observations that link dTMP biosynthesis to genome integrity in both prokaryotes and eukaryotes (reviewed in [47]). In this context, PLP deficiency may either cause aberrant uracil incorporation into DNA, generate a nucleotide imbalance that impairs DNA replication fork stability, or both. We therefore sought to assess the involvement of PLP in dTMP biosynthesis by testing whether *bud16*Δ cells accumulate uracil nucleotides in their DNA. To do so, we employed a recently described modified aldehydic slot blot assay that detects abasic sites produced when isolated DNA is treated with a uracil glycosylase enzyme [48]. As shown in Figure 8B, strains lacking Pdxk (*bud16*Δ) accumulate uracil in their genome significantly more than their wild-type counterparts. This accumulation is likely to be biologically important, as it is in the same range as the uracil accumulation observed in cells deficient in uracil glycosylase (*ung1*Δ cells), the main enzyme dedicated to the removal of uracil in DNA (Figure 8C). Furthermore, the double *ung1*Δ *bud16*Δ mutant accumulates more uracil in its genome than either of the single mutants, suggesting that *UNG1* and *BUD16* function in separate pathways to prevent uracil incorporation into DNA. These results are therefore consistent with a model in which the *bud16*Δ mutation increases dUMP pools, thereby increasing the frequency of dUTP incorporation into DNA.

Accumulation of uracil in genomic DNA may lead to DSB accumulation and attendant genome instability via excision of uracil and production of excessive abasic sites. However, deletion of *UNG1* does not suppress the *bud16*Δ genome instability rate and in fact results in a GCR rate increase (Table 2). This result indicates that either uracil excision is not a major cause of DNA damage in cells with low PLP levels, that an alternative excision pathway is involved, or that it is the accumulation of uracil that poses a threat to replication fork progression. Alternatively, it is also possible that a nucleotide pool imbalance caused by dUTP accumulation is a source of replication stress in *bud16*Δ cells. If *bud16*Δ cells have a defect in maintaining nucleotide pools, they may display some form of sensitivity to hydroxyurea (HU) a ribonucleotide reductase inhibitor. As shown in Figure 8D, HU dramatically affects the growth of *bud16*Δ cells at all concentrations tested and also leads to inviability of *bud16*Δ at 0.2 M HU, as measured by a colony-forming assay (Table S3). In contrast and as discussed above, *bud16*Δ cells are resistant to MMS, a DNA alkylating agent that causes DNA replication stress by impeding replication fork progression [49] (Figure 5D). Therefore, *bud16*Δ cells are sensitive to the depletion of deoxyribonucleotides rather than to replication stress. From these results, we suggest that PLP deficiency triggers DNA lesions due to a nucleotide imbalance resulting from defects in dTMP biosynthesis.

## Discussion

### A Genome-Wide Screen for Suppressors of Chromosome Rearrangements at *ChrXV-L*

In this report, we describe a screen for suppressors of rearrangements at *ChrXV-L,* a locus producing chromosome aberrations primarily via BIR. Since this locus is different from the commonly used *ChrV-L* locus, we expected overlapping and distinct sets of genes with those already known to prevent rearrangements of *ChrV*. Indeed, comparison of the results of our screen with a similar screen undertaken using the *ChrV-L* reporter chromosome [50] identifies only two overlapping GCR suppressor genes, *RAD5* and *MRE11*. This lack of overlap between both screens indicates that neither screen was saturating or that both loci can identify distinct classes of genome stability regulators. Indeed, we observed that disruption of some genes (such as *SGS1*) potently affects the GCR rate at the *ChrXV-L* locus while having a much more modest effect at *ChrV* [51], indicating that some genes may specifically suppress BIR.

In addition to Pdxk (Bud16), most of the other genes identified in our screen are likely to prevent DNA replication stress, suggesting that an unbiased screen for GCR suppressors is a potentially fruitful means of discovering novel activities influencing DNA replication. In particular, deletion of *WSS1,* which encodes a potential protease acting in the SUMO pathway [52], is synthetic lethal with deletion of *SGS1,* the yeast RecQ homolog [53]. This result, coupled with the high GCR rate of the *wss1*Δ strain, suggests that Wss1, perhaps via its proteolytic activity, acts in the management of DNA replication forks to prevent their demise. Likewise, Esc2 likely participates in maintaining replication fork integrity and tolerance to replication stress, given the ascribed role of its fission yeast homolog Rad60 in these processes [35,54]. Perhaps more puzzling is our identification of *ZIP1*, a meiotic gene, and *RML2,* encoding a mitochrondrial ribosome component, as GCR suppressors. Therefore, this study revealed several novel GCR regulators, which may be part of several less well-understood GCR suppression pathways. It will be important to decipher whether the products of these genes do indeed participate in the maintenance of mitotic genome integrity.

### Yeast as a System to Study the Influence of Micronutrients on Genome Stability

The link between decreased intracellular PLP levels and genome stability is important, since vitamin B6 deficiency correlates with heightened cancer risk [18–23]. This work therefore provides support for a model whereby subnormal levels of vitamin B6 may promote cancer development by engendering DNA lesions and attendant genome rearrangements. Given the poorly understood link between diet and cancer incidence, the *ChrXV-L* GCR assay provides a simple genetic system to probe the consequences of micronutrient deficiency on genome stability. Although the potential link between vitamin B6 and chromosome breakage had been suggested previously [20], the lack of a genetically tractable system to study the role of micronutrients in genome integrity has prevented a definitive mechanistic explanation of the vitamin B6–cancer epidemiological link. This situation has led to a multitude of alternative explanations. For example, other groups have contended that PLP decreases cellular proliferation or protects cells from oxidative stress [55,56]. We directly tested the possibility that reactive oxygen species affect the GCR rate of *bud16*Δ cells by growing them in the presence of the reactive oxygen species scavenger N-acetylcysteine (NAC). To our surprise, treatment with this compound increased rather than decreased the *bud16*Δ GCR rate (Table 2), indicating that reactive oxygen species may not play a major role in the formation of genome rearrangements when PLP levels are low.

### A Physiological Role for Intracellular Micronutrient Depletion?

Replication stress is thought to be a major deleterious event, as it is a source of DNA lesions and genome rearrangements. Paradoxically, recent observations point to a beneficial role for replication stress as an innate defense mechanism against tumorigenesis. Indeed, replication stress appears to be a hallmark of precancerous and hyperproliferating cells [57,58]. In this context, replication stress leads to activation of a DNA damage response that initiates senescence, thereby stopping the growth of a potential tumor [57–61]. These observations suggest that cells are wired to produce DNA lesions when their proliferation is aberrantly stimulated. One key and unresolved question that emerges from this body of work pertains to the nature of the cellular processes that trigger replication stress in response to uncontrolled cell growth. We speculate that our work, which links depletion of PLP to replication-associated DNA lesions, provides a simple mechanism that could link hyperproliferation to the activation of the DNA damage response. Indeed, we hypothesize that the exhaustion of metabolites through unscheduled anabolic processes may be primarily sensed as DNA replication stress. It will therefore be interesting to see whether intracellular PLP levels are decreased in precancerous lesions or whether Pdxk inhibition can sensitize cells to oncogene-induced cellular senescence.

## Materials and Methods

### Plasmid construction.

The *CAN1* gene from strain BY4741 was amplified from genomic DNA and cloned next to the *URA3MX* gene marker in the BglII site of pAG60 [62] to yield DDp418. To construct pBUD16 (DDp626), the *BUD16* locus encompassing the *BUD16* open reading frame was amplified by PCR from yeast genomic DNA and cloned in pCR2.1-TOPO (Invitrogen, http://www.invitrogen.com) and sequenced. The *BUD16* locus was then excised with SpeI and NotI and cloned into the SpeI and NotI sites of pRS415.

### Strain construction.

To construct the GCR assay strain (DDY643) the *CAN1* gene of BY4741 strain was replaced with the *MFA1pr-HIS3* marker [16]. This strain was then transformed with a PCR fragment containing the cycloheximide-resistance *cyh2* marker to yield strain DDY642. The *CAN1-URA3* cassette was amplified from DDp418 with primers *CAN1-URA3 F1:* 5′-GAA TCT GCC GTT TCG ATT TAC TTC GAT AAA GTT TGC GTT GTG AGT CAT ACG GCT TTT TTG-3′ and *CAN1-URA3 JM R1:* 5′-GGA AAA TTC TGG TCT ATT CAC AAT GAC AAG CGG TGA GCG TGT ATA GCG ACC AGC ATT CAC-3′ (underlined regions anneal to DDp418 and flanking regions are homologous to Chr*XV-L*). A second round of PCR with the following primers extended homology to the Chr*XV-L* region: *CAN1-URA3 F2:* 5′-TAT TGT GAA TTG AAA TTT AAA GTT ATC TCA AAT TCA AAT GAA TCT GCC GTT TCG ATT TAC-3′ and *CAN1-URA3 R2*: 5′-AGA TGG CTT TTC CAT CAG AGC CAT TGT GAA GAA ATC GGA GGA AAA TTG TGG TCT ATT CAC-3′ (underlined regions anneal to the PCR product from the first round). This amplified fragment was introduced in DDY642 to yield DDY643 and was verified by PCR analysis. The *MAT*α strain (DDY644) used in the screen was derived from DDY643 by mating type switching. All other strains were generated using genetic crosses, via one-step disruptions or via transformation of the indicated plasmids (see Table 4 for genotypes).

**Table 4:**
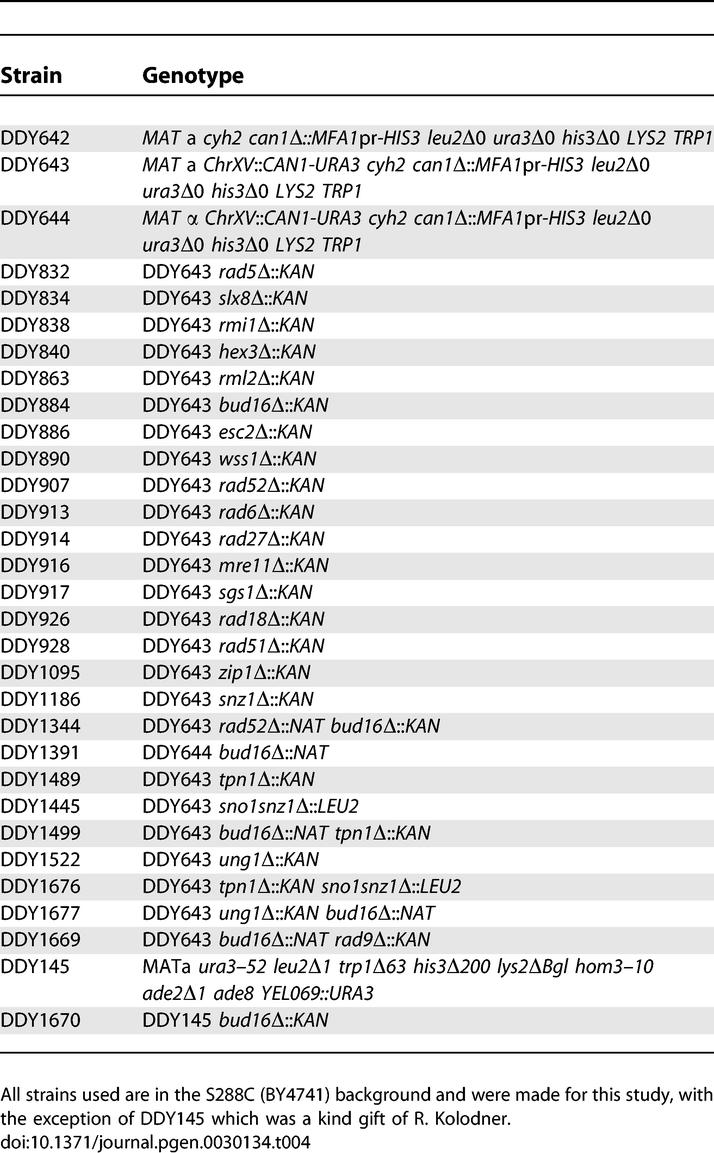
Yeast Strains Used in This Study

### Screening procedure.

To generate gene deletions in the *ChrXV-L* GCR assay strain, we employed synthetic genetic array technology, essentially as described by Tong et al. [16]. Briefly, DDY644 was mated to the *MATa* deletion strains from EUROSCARF (http://web.uni-frankfurt.de/fb15/mikro/euroscarf/) on YPD and incubated at 30 °C overnight. Diploids were selected on SD-URA + 200 mg/L G418 and incubated at 30 °C for 2 d. Sporulation proceeded on YE + 0.05% glucose for 7 d at 25 °C. Once sporulated, haploids were selected by a four-step pinning procedure: two selections on SD-URA-HIS + cycloheximide (10 mg/L) followed by two selections on SD-URA-HIS + cycloheximide (10 mg/L) + G418 (200 mg/L). Following the fourth selection step, each deletion mutant was hand patched onto nonselective rich XY media (2% peptone, 1% yeast extract, 0.01% adenine, and 0.02% tryptophan). Fourteen mutants in duplicate were patched onto a single 10-cm plate. They were allowed to grow for 2 d at 30 °C and were then replica plated onto agar plates to remove excess cells prior to replica plating onto FC (5-FOA and can) media. FC plates were incubated for 3 d at 30 °C following replica plating and analyzed for colony formation. Wild-type strains produced between 0–3 colonies on average. Therefore, we scored a patch as a positive hit if the threshold number of colonies per patch were equal to or greater than ten. Deletion mutants (1,160) were then placed in a “1 hit” category (for those with one patch that displayed greater than ten colonies) or were in a “2 hit” category (for those with both patches that showed greater than ten colonies). The “2 hit” category list, which consisted of 273 mutants, was narrowed down by focusing on genes that are expressed in the nucleus [31]. However, we did not discard any gene deletion that had unknown localization data. These filters reduced the number of positive hits to 125. Of these 125, we reconstructed 48 deletions in the DDY643 background. Once constructed and confirmed, these mutants were frozen at −80 °C immediately in SC-URA to ensure retention of the *CAN1-URA3* markers.

### Measurement of GCRs.

Strains were grown in SC-URA media to select for the *URA3-CAN1 ChrXV-L* arm prior to streaking cells onto nonselective rich media (XY). Single colonies were isolated and grown in 5 ml of XY medium until saturation. For wild-type strains, 1 ml of culture was spun down and plated onto a 10-cm FC plate and the number of cells/ml was calculated. A fluctutation test and the method of the median [24] was used to assess GCR rate. Similarily, for the *ChrV-L* GCR assay, a single colony was inoculated into 15 ml of XY medium until saturation. These cells were spun down and plated onto a 15-cm FC plate and the number of cells/ml was calculated. Again, a fluctuation test and the method of the median were used to measure GCR rates. To assess the effects of pyridoxine supplementation on genome stability, cells were grown in the absence or presence of 2 μg/ml pyridoxine hydrochloride (Supelco; http://www.sigmaaldrich.com/).

### Measurement of PLP by HPLC.

Wild-type, *bud16*Δ, *tpn1*Δ, *sno1snz1*Δ, and *tpn1*Δ *sno1snz1*Δ strains were grown in 100-ml cultures of XY + 2% glucose (with the exception of *tpn1*Δ *sno1snz1*Δ) to early log phase (OD_600_ 1.0). The cells were spun down and washed thrice with 50 ml of cold double-distilled water to remove any external PLP. Cells were then pelleted and lysed by glass beads in 5% TCA. Lysates were clarified by centrifugation at high speed to remove cell debris. PLP measurements were done blindly at the diagnostic division of Anticancer (http://www.anticancer.com/).

### Rad52-YFP fluorescent microscopy.

We assessed Rad52-YFP focus formation assay essentially as described by Lisby et al. [38] with the following modifications. Three independent isolates of each strain containing the p*RAD52*-*YFP* plasmid (a gift of Grant Brown) were grown in SC-LEU. Cells were imaged on an Nikon Eclipse E600 FN microscope (http://www.nikonusa.com) equipped with an ORCA ER2 camera (http://www.hamamatsu.com) and Chroma filters (http://www.chroma.com/). Micrographs were taken in 21 *z*-stacks with 0.007-μm increments. For each independent isolate, a minimum of 180 cells were examined.

### Mammalian cell culture.

HeLa cells were grown in DMEM supplemented with 10% fetal calf serum. SiHa cervical carcinoma cells were obtained from the American Type Culture Collectin (http://www.atcc.org) and maintained in exponential growth by twice-weekly subcultivation in minimal essential medium containing 10% fetal bovine serum (Gibco, http://www.invitrogen.com/). A stock solution of 4-DP (200 mM) was prepared in 0.9% saline, diluted in growth medium, and adjusted to pH 7.2.

### Western blotting and kinase assays.

Human whole-cell extracts (25 μg) were prepared by boiling the cellular pellet in 1× SDS sample buffer for 5 min. Extracts were loaded onto an SDS-PAGE gel and after electrophoresis, proteins were transferred to a PVDF membrane (Millipore, http://www.millipore.com/) and immunoblotted with either the phospho-Chk2(Thr68) or Chk2 primary antibodies (Cell Signaling Technology, http://www.cellsignal.com/) followed by horseradish peroxidase-coupled secondary antibody (Jackson ImmunoResearch Laboratories, http://www.jacksonimmuno.com/). Rad53 immunoblotting and autokinase assays were carried out on denatured cell extracts exactly as described previously [9,63].

### Immunofluorescence.

HeLa cells grown on coverslips were washed twice in PBS and fixed with 2% PFA for 1 h at room temperature, washed, and permeabilized with 1% Triton-X in PBS (1h, room temperature) and subsequently blocked for an additional 1 h in 10% antibody dilution buffer (10% normal goat serum, 3% BSA, and 0.05% Triton X in PBS). Monoclonal 53BP1 antibody (Transduction Laboratories) was diluted in blocking buffer and incubated with cells overnight at room temperature followed by two 10 min washes in 0.05% Triton X-100 in PBS. The appropriate Alexa-555 conjugated secondary antibody (Molecular Probes, http://probes.invitrogen.com/) was diluted 1:500 in 10% antibody dilution buffer and incubated with the coverslips for 2 h at room temperature. After several washes with PBS, the cells were stained with DAPI (10 μg/ml) for 20 min and mounted with ProLong Gold anti-fade agent (Molecular Probes).

### Pulse-field gel electrophoresis.

Briefly, ∼1 × 10^8^ cells grown from a saturated culture were spun down, washed, and then resuspended in TE (pH 7.5) and Zymolyase (Zymo Research, http://www.zymoresearch.com. Plugs were formed by mixing liquefied low melt agarose (SeaKem; http://www.lonza.com) with the resuspended cells and solidified in plug molds. Plugs were transferred into LET solution (0.5M EDTA pH 8.0, 0.01M Tris-HCl (pH 7.5), 40mM DTT, and 0.4mg/ml Zymolyase) overnight at 37 °C. Plugs were then transferred into fresh tubes containing NDS solution (0.5 M EDTA pH 9.5, 0.01 M Tris-HCl pH 7.5, 1% N-lauroyl sarcosine sodium salt, and 2 mg/ml proteinase K) and incubated at 50 °C overnight. Plugs were washed several times in TE (pH 7.5) and incubated for 1 h. Plugs containing whole chromosomes were immediately run on a CHEF-DR III system (Bio-Rad, http://www.bio-rad.com/) using a 1% agarose gel and 0.5× TBE at 14 °C, switch time 6–120 s, angle 120° for 24 h with a voltage gradient of 6V/cm. To examine the size of the terminal restriction fragment on *ChrXV-L,* whole chromosomes were prepared as described above in agarose plugs and were then digested with the restriction enzyme PmeI. Plugs of digested chromosomes were run on a 1% agarose gel in 0.5× TBE at 14 °C, switch time 1–15 s, angle 120°, 19 h with a voltage gradient of 6V/cm.

### Flow cytometry and cell size.

SiHa cells (5 × 10^5^) were fixed in 1.4 ml of 70% ethanol and kept at −20° C for up to two weeks before analysis. All fixed samples were prepared for antibody staining and analyzed on the same day. One milliliter of cold Tris-buffered saline, pH 7.4 (TBS) was added to each tube, then the cells were spun down and resuspended in 1 ml of cold 4% FBS and 0.1% Triton X-100 (TST) and placed on ice. Cells were allowed to rehydrate for 10 min, then spun down and resuspended in 200 μl of mouse monoclonal anti-phospho-histone H2A.X antibody (Upstate Biotechnology http://www.millipore.com/), which was diluted 1:500 in TST. Tubes were incubated on a shaker for 2 h at room temperature, rinsed with cold TST, and resuspended in 200 ul of secondary antibody (Alexa 488 goat antimouse IgG [H + L]F[ab′]2 fragment conjugate [Molecular Probes] diluted 1:200 in TST) for 1 h at room temperature. Cells were rinsed in 2% FBS in TBS and resuspended in 400 μl of cold TBS containing 1 μg/ml DAPI. Samples were analyzed using a Coulter Elite dual laser flow cytometer (http://www.beckmancoulter.com). List mode files were analyzed using WinList software (Verity Software House, http://www.vsh.com/).

### Uracil detection.

DNA was isolated from yeast strains using Qiagen (http://www.qiagen.com) gravity tip columns as per the manufacturer's protocol and assayed for uracil incorporation as described in Cabelof et al. [64]. Briefly, 4 μg of DNA was blocked for 2 h at 37 °C in a 2× tris/methoxyamine buffer (final concentration: 100 mM methoxyamine [Sigma-Aldrich, http://www.sigmaaldrich.com/] and 50 mM Tris-HCl, pH 7.4). DNA was precipitated with 7.5% volumes of 4 M NaCl and 4 volumes of ice-cold 100% ethanol and resuspended in TE buffer, pH 7.6. DNA was then treated with 0.4 units of Uracil DNA Glycosylase (New England Biolabs, http://www.neb.com) for 15 min at 37 °C, immediately precipitated, and resuspended in TE buffer, pH 7.6. DNA was then probed with 2 mM aldehydic reactive probe (Dojindo Molecular Technology, http://dojindo.com/) for 15 min at 37 °C followed by ethanol precipitation and resuspension in TE buffer, pH 7.6. DNA was then heat denatured, immobilized onto a nitrocellulose membrane (Schleicher and Schuell, http://www.whatman.com/), and baked under vacuum as originally described by Nakamura et al. [48]. The dried membrane was washed in 5× SSC for 15 min at 37 °C, then incubated in a prehybridization buffer (20 mM Tris, pH 7.5; 0.1 M NaCl; 1 mM EDTA; 0.5% casein w/v; 0.25%BSA w/v; and 0.1% Tween-20 v/v) for 30 min at room temperature. Streptavidin-POD conjugate (Roche, http://www.roche.com/) was added at a 1:2,000 dilution for 45 min at room temperature. Membrane was washed in TBS/Tween-20 three times at 37 °C, incubated in ECL solution (Pierce, http://www.piercenet.com/) for 5 min at room temperature, then visualized and quantified using a ChemiImagerTM system (Alpha Innotech, http://www.alphainnotech.com/). Data are expressed as the integrated density value of the band per microgram of DNA loaded on the membrane.

### Comparative genome hybridization.

Yeast genomic DNA was prepared from saturated 10-ml cultures essentially by the method of [65]. Genomic DNA (2 μg) was digested for 2 h with 10 U of HaeIII and purified by phenol-chloroform extraction and ethanol precipitation. HaeIII-digested genomic DNA (50 μg) was labeled and hybridized by the method of [66]. Briefly, after blunting the DNA ends with T4 polymerase, the fragments were ligated to unidirectional linkers and amplified by ligation-mediated PCR in the presence of aminoallyl-modified dUTP. Indirect labeling was performed using monoreactive Cy5 (for the parental strain) or Cy3 (strains that had undergone GCR) NHS esters that react specifically with the aminoallyl-dUTP. Control and experimental samples were combined, and the labeled DNA was hybridized to a yeast whole-genome ChIP-on-chip microarray (4 × 44K; Agilent Technologies, http://www.home.agilent.com/) and scanned at the University Health Network Microarray Centre (http://www.microarrays.ca/). Microarray images were processed with GenePix Pro 6.0 (Molecular Devices, http://www.moleculardevices.com). Data were analysed as described previously [67]. Hybridization data were preprocessed with ArrayPipe 1.7 [68], the background was subtracted using the “foreground-background” correction method, the data were normalized using the “linear model for microarray analysis (limma) loess (subgrid) method,” and the results were mapped using the University of California Santa Cruz genome browser (http://genome.ucsc.edu/cgi-bin/hgGateway).

Text S2 contains supplementary materials and methods for apoptosis analysis, yeast DNA content, and cell size distributions.

## Supporting Information

Figure S1 A Semi-Quantitative Papillation-Based Assay to Estimate GCR Rates (6.5 MB PDF)

Figure S2 Cell Cycle Progression and Cell Size Phenotypes of *bud16*Δ (439 KB PDF)

Figure S3 4-DP Does Not Trigger Apoptosis (479 KB PDF)

Table S1 Growth Rate of Vitamin B6 Mutants (31 KB DOC)

Table S2 Mean and Median Cell Size of *bud16*Δ and *tpn1*Δ Mutants (32 KB DOC)

Table S3 Cell Viability and Growth Rate of *bud16*Δ Mutants Grown in HU and MMS (38 KB DOC)

Text S1 Supplementary Results Phenotypic characterization of the *bud16*Δ mutant.
(39 KB DOC)

Text S2 Supplementary Materials and Methods Apoptosis analysis, yeast DNA content, and cell size distributions.
(33 KB DOC)

## Abbreviations

BIR: break-induced replication
can: canavinine
*ChrV-L,*: left arm of Chromosome *V*
DSB: double-strand breaks
4-DP: 4-deoxypyridoxine
FC: 5-fluoro-orotic acid and canavinine; 5-fluoro-orotic acid
GCR: gross chromosomal rearrangement
HR: homologous recombination
HU: hydroxyurea
MMS: methyl methanesulfonate
NAC: N-acetylcysteine
Pdxk: pyridoxal kinase
PFGE: pulsed-field gel electrophoresis
PLP: pyridoxal 5′ phosphate
YFP: yellow fluorescent protein
