# Supplementary material for: A Screen for Suppressors of Gross Chromosomal Rearrangements Identifies a Conserved Role for PLP in Preventing DNA Lesions
Source: PLoS Genet. 2007 Aug 10;3(8):e134. doi: 10.1371/journal.pgen.0030134 (PMC1941753; doi:10.1371/journal.pgen.0030134)
Supplement: Supplementary file 3 [file pgen.0030134.sg003.pdf]

No treatment

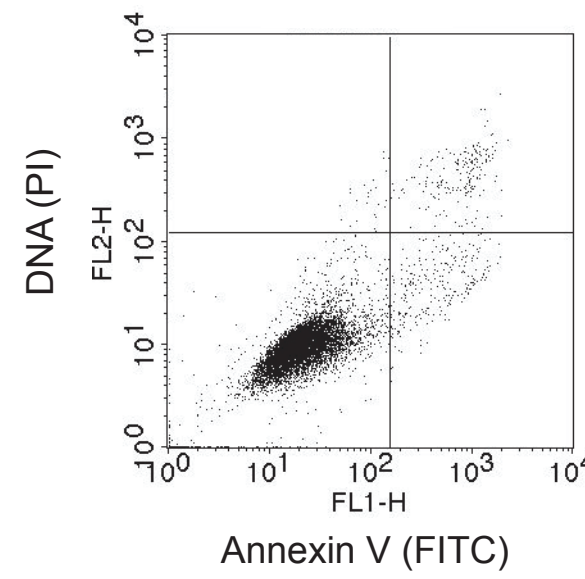

Apoptotic cells: 1.9 %

10 mM 4-DP

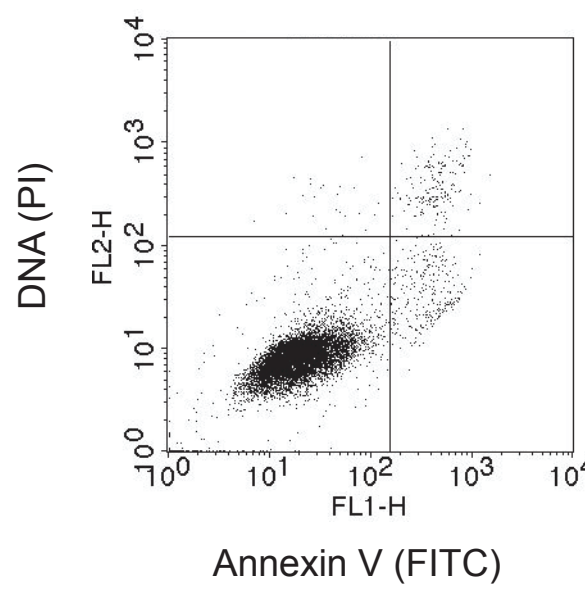

Apoptotic cells: 1.8 %

25 μM etoposide

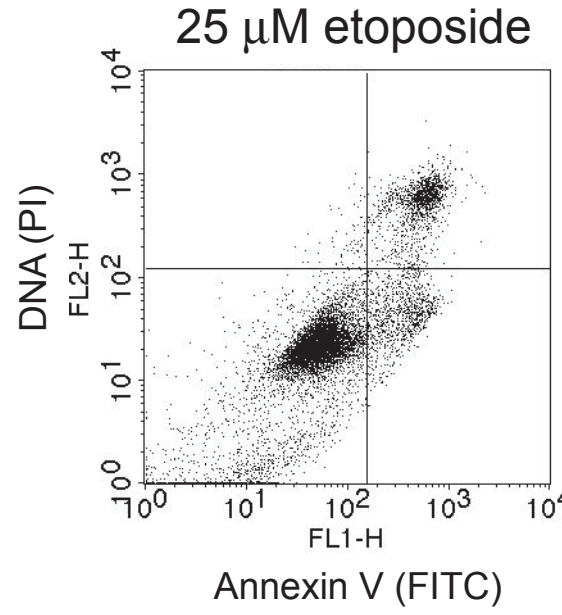

Apoptotic cells: 13.8 %

Figure S3: Kanellis *et al.*
