# Supplementary material for: A Screen for Suppressors of Gross Chromosomal Rearrangements Identifies a Conserved Role for PLP in Preventing DNA Lesions
Source: PLoS Genet. 2007 Aug 10;3(8):e134. doi: 10.1371/journal.pgen.0030134 (PMC1941753; doi:10.1371/journal.pgen.0030134)
Supplement: Supplementary file 4 [file pgen.0030134.sd001.doc]

# SUPPLEMENTARY MATERIAL

**SUPPLEMENTARY RESULTS**

***Phenotypic characterization of the* *bud16*Δ *mutant***

To better characterize the spectrum of phenotypes associated with the *bud16Δ* mutation, we analyzed its growth properties, cell cycle profile and cell size characteristics. First, we monitored the growth of *bud16Δ* in rich medium. As shown in Table S1 *bud16Δ* exhibits a doubling time of 4.5 ± 0.4 hrs compared to 1.5 ± 0.3 hrs for the wild-type strain grown under the same conditions. *bud16Δ* cells grow at all temperatures tested and thus the mutant does not appear to be temperature-sensitive (data not shown). Next, we monitored the cell cycle distribution of asynchronously cultured *bud16Δ* cells as a means to identify the basis of its slow growth. We first analyzed the DNA content of wild-type and *bud16Δ* cultures by flow cytometry. *bud16Δ* cultures show an accumulation of cells at the G1 phase of the cell cycle (Figure S2A). Given the role of PLP in amino acid and nucleotide metabolism, we reasoned that this accumulation in G1 could either be the result of a sensed nutrient deficiency (i.e. cells have difficulty reaching START) or may be the result of a checkpoint arrest. We therefore deleted *RAD9*, a checkpoint gene in the *bud16Δ* background and examined the DNA content of *bud16Δ rad9Δ* cultures by flow cytometry. As shown in Figure S2A, the cell cycle profile of *bud16Δ rad9Δ* cultures is undistinguishable from the profile of *bud16Δ* cells indicating that a DNA damage checkpoint is not the cause of the G1 accumulation.

We next examined cell cycle progression following the release from G1 synchronization. As shown in Figure S2B, *MAT***a** *bud16Δ* cells can be readily synchronized by the alpha-factor pheromone, although it is not certain, given its slow growth, whether all or only a subset of *bud16Δ* cells reach the pheromone arrest point. Although removal of the alpha-factor from the growth media allows wild-type cells to initiate and complete DNA replication within 60 min, *bud16Δ* cells display a mixed phenotype with a fraction of cells able to proceed with DNA replication with normal kinetics while a majority of cells appear arrested in G1. Since *bud16Δ* cells do not appear hypersensitive to alpha-factor (data not shown), these results rather suggest that *bud16Δ* cells accumulate in G1 phase due to a sensed nutrient deficiency caused by decreased intracellular PLP levels.

In order to ascertain whether the G1 accumulation of *bud16Δ* cultures is typical of defects in the vitamin B6 salvage pathway, we also examined the DNA content of *tpn1Δ* cells by flow cytometry. As shown in Figure S2A, *tpn1Δ* cells show a G1 accumulation phenotype similar to that of *bud16Δ* cells. The cell cycle distribution of the *tpn1Δ bud16Δ* double is similar to that of *bud16Δ* cultures, consistent with both genes acting within the same biological pathway (Figure S2A). Finally, we noted during the course of our studies that *bud16Δ* cells display small mean and median cell sizes, consistent with its G1 accumulation (Figure S2C and Table S2). *tpn1Δ* cultures also display small mean and median cell size as compared to wild-type cells (Figure S2C and Table S2). Accordingly, *bud16Δ tpn1Δ* cultures display the same mean and median cell sizes as either single mutant, consistent with their epistatic relationship.

**Supplemental Figure Legends**

Figure S1**.** **The semi-quantitative papillation-based assay to estimate GCR rates**.

(A), Individual colonies of the indicated genotype (in the *ChrXV-L* GCR assay background) were constructed and patched on non-selective rich media and then replica-plated on media containing 5-FOA and canavinine (FC). All four strains increase GCR rates when compared to the parent strain (top and bottom rows) and display reproducible increased papillation on FC plates. The detailed procedure of the GCR screen can be found in the Materials and Methods.

(B) Raw data from the screen indicating positive hits patched in duplicate. Boxed patches correspond to known GCR mutator strains and *bud16Δ* (outlined with a red box). XY is a rich media.

Figure S2. **Cell cycle progression and cell size phenotypes**.

(A) Cultures of *bud16Δ*, *tpn1Δ* and *tpn1Δ bud16Δ* all display a large proportion of cells in the G1 phase of the cell cycle, as measured by FACS profiles.

(B) Wild-type and *bud16Δ* cells were synchronized with alpha-factor and then released (t=0) to monitor cell cycle progression. *ASN* refers to asynchronous cell population prior to cell synchronization.

C) Cell size distributions of the indicated strains as assessed on a Coulter channelizer.

Figure S3. **Treatment of cells with 4-DP do not trigger apoptosis**. Shown are flow cytometic profiles of cells treated with the indicated drugs for 48 hrs. Cells were then processed for Annexin V and propidium iodide staining. Cells in the upper-right quadrant correspond to apoptotic cells.
