# Supplementary material for: A Screen for Suppressors of Gross Chromosomal Rearrangements Identifies a Conserved Role for PLP in Preventing DNA Lesions
Source: PLoS Genet. 2007 Aug 10;3(8):e134. doi: 10.1371/journal.pgen.0030134 (PMC1941753; doi:10.1371/journal.pgen.0030134)
Supplement: Supplementary file 5 [file pgen.0030134.sd002.doc]

**SUPPLEMENTARY MATERIALS AND METHODS**

**Apoptosis analysis, yeast DNA content and cell size distributions**

For the analysis of apoptosis, we analyzed Annexin V/propidium iodide staining on a BD FACScalibur instrument. For flow cytometry of DNA content in yeast, we carried out essentially the same protocol as described in (Kanellis et al. 2003) using SYBR Green (Molecular Probes). Cell size distributions were measured as described in (Jorgensen et al. 2002) from log phase cells grown to ~0.3-3x10^7^ cells/mL. Cells were gently sonicated and the mean and median cell size was measured on a Coulter Channelizer Z2 (Beckman-Coulter). Values of the mean and median cell sizes can be found in Table S2.

**Supplementary References**

Jorgensen, P., Nishikawa, J.L., Breitkreutz, B.J., and Tyers, M. 2002. Systematic identification of pathways that couple cell growth and division in yeast. *Science* **297**(5580): 395-400.

Kanellis, P., Agyei, R., and Durocher, D. 2003. Elg1 forms an alternative PCNA-interacting RFC complex required to maintain genome stability. *Curr Biol* **13**(18): 1583-1595.
