# Supplementary material for: A Screen for Suppressors of Gross Chromosomal Rearrangements Identifies a Conserved Role for PLP in Preventing DNA Lesions
Source: PLoS Genet. 2007 Aug 10;3(8):e134. doi: 10.1371/journal.pgen.0030134 (PMC1941753; doi:10.1371/journal.pgen.0030134)
Supplement: Supplementary file 6 [file pgen.0030134.st001.doc]

Table S1 : Growth rate of vitamin B6 mutants

| **Strain** | **Doubling Time at 30ºC (hrs) ± S.D.** |
| --- | --- |
| *wildtype* | 1.5 ± 0.3 |
| *snz1sno1*Δ | 1.5 ± 0.3 |
| *tpn1*Δ | 3.1 ± 0.1 |
| *bud16*Δ | 4.5 ± 0.4 |
| *tpn1*Δ *snz1sno1*Δ | 4.9 ± 0.9 |
| *tpn1*Δ *snz1sno1*Δ + 2μg/ml pyridoxine | 1.8 ± 0.2 |
