# Supplementary material for: A Screen for Suppressors of Gross Chromosomal Rearrangements Identifies a Conserved Role for PLP in Preventing DNA Lesions
Source: PLoS Genet. 2007 Aug 10;3(8):e134. doi: 10.1371/journal.pgen.0030134 (PMC1941753; doi:10.1371/journal.pgen.0030134)
Supplement: Supplementary file 7 [file pgen.0030134.st002.doc]

Table S2 :Mean and Median cell size of *bud16*Δ and *tpn1*Δ mutants

| **Strain** | **Mean Cell Size (fL) ± S.D.** | **Median Cell Size (fL)** |
| --- | --- | --- |
| *wildtype* | 45.9 ± 1.7 | 47.5 |
| *bud16*Δ | 39.2 ± 1.7 | 41.7 |
| *tpn1*Δ | 38.9 ± 1.9 | 40.7 |
| *bud16*Δ *tpn1*Δ | 39.2 ± 1.8 | 41.0 |
