# Supplementary material for: A Screen for Suppressors of Gross Chromosomal Rearrangements Identifies a Conserved Role for PLP in Preventing DNA Lesions
Source: PLoS Genet. 2007 Aug 10;3(8):e134. doi: 10.1371/journal.pgen.0030134 (PMC1941753; doi:10.1371/journal.pgen.0030134)
Supplement: Supplementary file 8 [file pgen.0030134.st003.doc]

Table S3: Cell viability and growth rate of *bud16*Δ mutant grown in HU and MMS

|  | *Wild-type* | | *bud16Δ* | |
| --- | --- | --- | --- | --- |
| Treatment | Percent Viability (% ± S.E.M.) | Length of Growth (days) | Percent Viability (% ± S.E.M.) | Length of Growth (days) |
| No treatment | 100 ± 8 | 2 | 100 ± 8 | 3 |
| 0.05 M HU | 124 ± 19 | 2 | 71 ± 11 | 3 |
| 0.1 M HU | 117 ± 8 | 3 | 59 ± 11 | 5 |
| 0.15 M HU | 107 ± 11 | 3 | 54 ± 2 | 6 |
| 0.2 M HU | 107 ± 11 | 3 | 0 ^1^ | 8 |
| 0.005% MMS | 103 ± 5 | 2 | 84 ± 16 | 3 |
| 0.01% MMS | 99 ± 7 | 2 | 104 ± 5 | 3 |
| 0.02% MMS | 99 ± 14 | 3 | 71 ± 6 | 4 |

^1^ No colonies were observed after 8 days of growth at 30ºC.
